# High-throughput phenotyping to dissect genotypic differences in safflower for drought tolerance

**DOI:** 10.1371/journal.pone.0254908

**Published:** 2021-07-23

**Authors:** Sameer Joshi, Emily Thoday-Kennedy, Hans D. Daetwyler, Matthew Hayden, German Spangenberg, Surya Kant

**Affiliations:** 1 Agriculture Victoria, Grains Innovation Park, Horsham, Victoria, Australia; 2 Agriculture Victoria, AgriBio, Centre for AgriBioscience, Bundoora, Victoria, Australia; 3 School of Applied Systems Biology, La Trobe University, Bundoora, Victoria, Australia; 4 Centre for Agricultural Innovation, School of Agriculture and Food, Faculty of Veterinary and Agricultural Sciences, The University of Melbourne, Melbourne, Victoria, Australia; Harran Universitesi, TURKEY

## Abstract

Drought is one of the most severe and unpredictable abiotic stresses, occurring at any growth stage and affecting crop yields worldwide. Therefore, it is essential to develop drought tolerant varieties to ensure sustainable crop production in an ever-changing climate. High-throughput digital phenotyping technologies in tandem with robust screening methods enable precise and faster selection of genotypes for breeding. To investigate the use of digital imaging to reliably phenotype for drought tolerance, a genetically diverse safflower population was screened under different drought stresses at Agriculture Victoria’s high-throughput, automated phenotyping platform, Plant Phenomics Victoria, Horsham. In the first experiment, four treatments, control (90% field capacity; FC), 40% FC at initial branching, 40% FC at flowering and 50% FC at initial branching and flowering, were applied to assess the performance of four safflower genotypes. Based on these results, drought stress using 50% FC at initial branching and flowering stages was chosen to further screen 200 diverse safflower genotypes. Measured plant traits and dry biomass showed high correlations with derived digital traits including estimated shoot biomass, convex hull area, caliper length and minimum area rectangle, indicating the viability of using digital traits as proxy measures for plant growth. Estimated shoot biomass showed close association having moderately high correlation with drought indices yield index, stress tolerance index, geometric mean productivity, and mean productivity. Diverse genotypes were classified into four clusters of drought tolerance based on their performance (seed yield and digitally estimated shoot biomass) under stress. Overall, results show that rapid and precise image-based, high-throughput phenotyping in controlled environments can be used to effectively differentiate response to drought stress in a large numbers of safflower genotypes.

## Introduction

Safflower (*Carthamus tinctorius* L) is one of the oldest oilseed crops, currently grown in more than 25 countries, with Kazakhstan, USA, Mexico and India being major producers in 2018 [1]. Safflower is believed to be domesticated about 4,000 years ago in the Fertile Crescent and has various centres of origins such as India-Pakistan, North Africa, Middle East, and Europe [2]. The Safflower accessions from Iran, India, Turkey and Pakistan were genetically similar having grouped together while the genotypes from Afghanistan and China were distinct and hence grouped in a different cluster [3]. The safflower accessions within the proposed centres were related to one another for parameters such as height, flower colour and branching while variations were observed between the centres of origin [4]. Due to its drought stress tolerant nature, safflower is a suitable crop for dryland agriculture [5]. Safflower is an undervalued yet highly versatile crop, with young plants consumed as a vegetable, petals used to produce the dye carthamin, and seed oil used in various industries [5–7]. Apart from its industrial value, safflower is used in traditional medicine across Asia and the Middle East [8]. Recently, with the preference for the plant-based renewable oil, there has been an increased demand for safflower oil, with its use in biofuel [9, 10], textile [10], food [11, 12], pharmaceutical [13] and cosmetic [14] industries. Safflower is an excellent source of oil, yielding 32–40% oil/g of meal, with significant varietal variations in fatty acid composition [15]. Safflower varieties could contain up to 70% polyunsaturated linoleic acid, as well as monounsaturated oleic acid and stearic acid in variable concentrations, suiting different industries [4, 16, 17]. Due to increased demand in the biofuel and biolubricant industries, recent breeding programs have been directed towards selection of superior safflower genotypes with over 75% oleic acid, a higher purity than any other oil seed crops [18–21].

In general, safflower is well adapted to cultivation in water limited environments. Forming strong and deeper root system, (1.6–2 m in depth) safflower can access water and nutrient reserves often unattainable for most crops [5, 22, 23]. The presence of xerophytic spines also indicate the drought and heat tolerant nature of safflower [5]. In fact, safflower is significantly susceptible to many soil and plant pathogens associated with wet conditions, with a particular aversion to wet soil during germination [5, 6]. With the predicted decrease in crop yields under changing climatic conditions [24], safflower is a potential saviour crop for dry land agriculture, due to its ability to produce reasonable grain yield under drought stress [23, 25–27]. Although, previous research has found considerable variability in growth, seed production and oil yields of different safflower genotypes when grown in arid field environments [25, 26, 28–34]. Most of the previous research into safflower drought tolerance has relied on in-field screening methods, which are time-consuming, laborious and subject to environmental variations. Therefore, the use of reliable, high-throughput glasshouse-based screening techniques is vital to identify drought tolerant safflower genotypes [35].

Drought is an unpredictable stress in terms of occurrence at timing (crop growth stage), duration and severity and adversely affecting crop production [36, 37]. Safflower, like most crops, is sensitive to extreme drought conditions throughout its lifecycle (Hussain et al. 2015), although reproductive stages are the most vulnerable to stress, where alterations of critical factor including photosynthetic efficiency and nutrient relocation affect yield and grain quality [38, 39]. Reductions in the availability of water during vegetative growth significantly affect chlorophyll content, membrane stability and leaf area which can also have an impact on yield [40]. During seed filling stages, drought has negative consequences for physiological parameters including leaf temperature, osmotic adjustment and stomatal conductance in safflower [31]. The drought impact can have severe effects with a reduction of up to 20% of oil yield and a substantial decrease in linoleic acid, and an increase in palmitic and stearic acid contents [41]. The strong association of oil yield with other morphological traits such as seed yield, number of capitula and number of branches [19] and the relationship between biomass with plant height and yield [42] provide important strategies to breed for morphological traits which in turn will improve the seed and oil yield. The association between several drought indices such as yield index [43], geometric mean productivity (GMP) [44], mean productivity (MP) [45], tolerance index (TOL) [45] and stress tolerance index (STI) [46] have been used previously as tools in screening and selecting drought tolerant genotypes in the breeding programs. Higher values of GMP, MP and STI [47], and lower values of TOL are preferred in selecting the drought tolerant genotypes [46]. Unfortunately, due to the complex nature of drought stress there is no consensus on the ideal method for screening germplasm for drought responses [48].

Currently, unavailability of accurate, reliable and high-throughput phenotyping technologies is a bottleneck in complementing the rapid developments in genotyping technologies that play an important role in high-speed crop breeding [49]. Traditional phenotyping methods are time and resource consuming, often including considerable variation and destructive harvesting, making it impossible to repeatably observe the same plant throughout its growth cycle [49]. The expansion of high-throughput phenotyping technologies, involving the use of digital, proximal imaging, and sensor developments, has allowed non-destructive, precise, rapid and repetitive measurements to be feasible [48]. High-throughput, image-based phenotyping can be perform from the landscape to the cellular level, using platforms such as satellites, unmanned aerial vehicles, vehicle mounted sensors, and hand-held cameras or sensors [50]. By measuring the interactions between plant components and the light spectrum, sensors can provide critical information regarding plant traits [50]. Using specific spectral regions of the electromagnetic spectrum, including visible/ red-green-blue (RGB; 400–700 nm) and near infra-red (700–1000 nm) regions, sensors and cameras can sense physiological and phenotypical changes [51]. Under controlled and field environments, high-throughput digital phenotyping has been employed to understand germination [52], estimated shoot biomass [53–55], early vigour [56, 57], root architecture [58], biomass at flowering [59], leaf morphology [60], detection of disease infection [61] and yield [62]. Non-destructive estimations of growth can produce high correlations between estimated shoot biomass, shoot fresh and dry weights in a range of crops [53, 54, 56, 63]. High-resolution, multi-time point measurements on the same plant enable the study of dynamic growth rates over time [54, 55, 64, 65]. Digital imaging coupled with image processing algorithms are key to efficiently dissecting plant traits, allowing neglected crops such as safflower to be explored for responses to stresses such as drought.

Here, we present the establishment and application of a non-destructive, automated, high throughput, image-based phenotyping protocol, using different watering regimes, for the precise controlled environment screening for drought tolerance in safflower. To our knowledge, this work represents the first reported application of digital image-based phenotyping to investigate drought response in safflower and to identify drought tolerant genotypes.

## Materials and methods

### Plant materials and experimental set up

**Fig 1:**
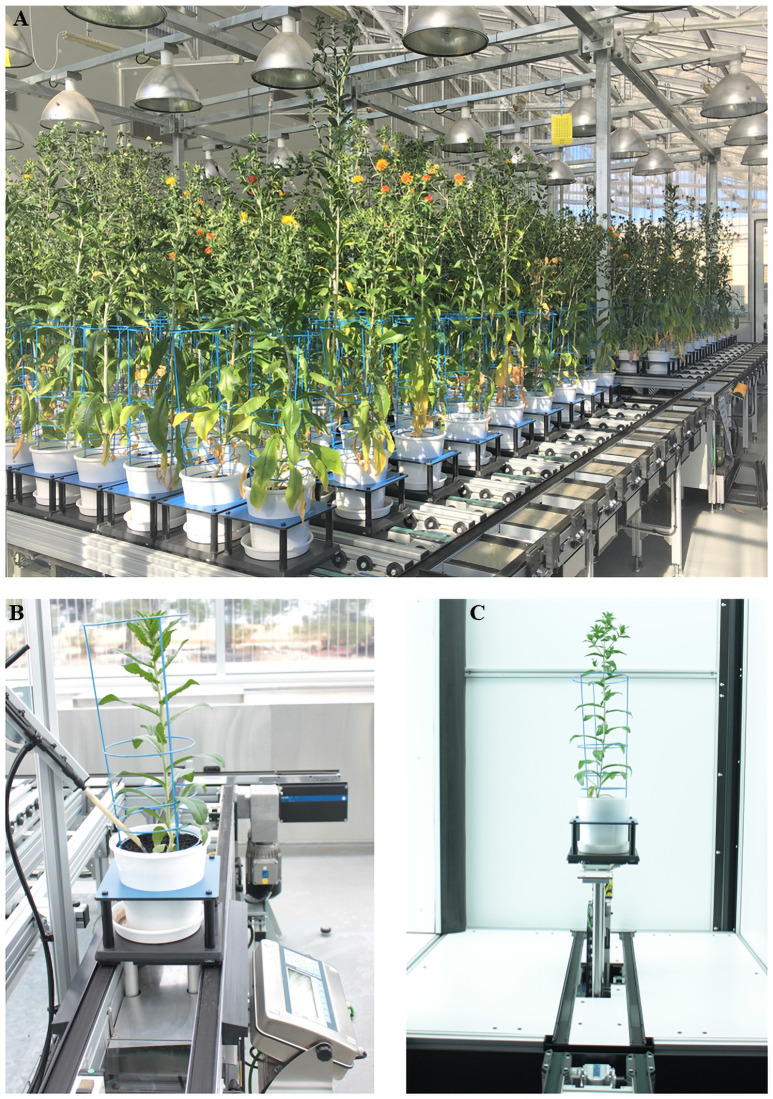
Safflower plants at different drought treatments, growing at Plant Phenomics Victoria, Horsham. (A) Plants in the white pots and carriers on the conveyor system during flowering stage. (B) Plant receiving water at an automated weighing and watering station. (C) Plant in the digital imaging cabinet during image acquisition.

Two separate experiments were conducted at Plant Phenomics Victoria, Horsham (PPVH), Agriculture Victoria’s state-of-the-art high-throughput phenotyping facility. In brief, PPVH contains two climate-controlled glasshouses holding 600 pots, fitted with conveyor systems and automated weighing and watering stations for precise water delivery, as well as a high resolution Scanalyzer 3D digital imaging platform (Fig 1; Lemnatec GmBH, Aachen, Germany). Detailed descriptions of the facility and cameras can be found in Banerjee et al [45].

In the first experiment, four released safflower (*Carthamus tinctorius* L.) genotypes having different oil types, Gila, Sironaria, (belong to linoleic oil type) [66], S317 [67] and Montola2003 [68] (belong to oleic oil type), were phenotyped to establish the optimal watering treatment to screen for drought response. Based on these results, 200 diverse safflower genotypes from the Agriculture Victoria safflower collection, chosen to represent maximum genetic diversity (S1 Table), which were screened in the second experiment to validate the developed protocol and identify drought tolerant genotypes.

Plants in both experiments were grown in 200 mm Euro-TL white pots (Garden City Plastics, VIC, Australia) filled with 3.25 L of standard potting mix (Biogro, SA, Australia). Added to 1000 L of standard potting mix were 3 kg Floranid N 32 IBDU (Compo GmbH & Co. KG, Münster, Germany), 5 kg Standard Brown Nutricote (Yates Australia, NSW, Australia), 3 kg Blue Coloniser Plus Macracote (Langley Fertilizer, WA, Australia), 1 kg MicroPlus Trace Element Fertiliser (Langley Fertilizer, WA, Australia), 225 g LiberFer SP Fe-chelate (BASF Corporation, NJ, USA) and 2 kg Debco SaturAid (Evergreen Garden Care Australia Pty Ltd, NSW, Australia) to ensure optimal growth and development of plants. Pots were weighed and equated to a uniform weight, lightly irrigated before sowing and placed on saucers to prevent water loss. Three seeds were sown per pot, then thinned to retain one seedling per pot of uniform vigour across experiment. Plants were supported using blue cages and loaded on to the conveyor system at 15 DAS and grown until maturity. Growth conditions were controlled at 24°C/15°C day/night.

### Determination of field capacity and watering regimes

The average field capacity (FC) per pot was determined using the soil gravimetric water content (SGWC) method [69, 70]. In brief, standard potting mix from eight pots was saturated with water and let drain until water stopped flowing, then the wet weight of the pot was recorded. The wet potting mix from each pot was dried separately in an oven at 70°C for 5 days, then the dry weight of potting mix was recorded. Data for eight pots per experiment was averaged to calculate the pot weight for 100% FC. Based on this value, the pot weights for 90%, 50% and 40% FC were calculated. Pot weight for each treatment was monitored daily throughout the growth cycle to maintain the specified FC and used to map stress levels as reported in previous research studies [69, 71]. Pot weights per day for each treatment, throughout the growing cycle, are presented in S1 Fig, which illustrates the four different treatment regimes. During the early growth stages pot weight for all treatments was maintained at 5200 g, then increased to 5300 g (90% FC) for control plants to take into consideration of the plant biomass during the initial vegetative growth period. The plants that were in the recovery phase were maintained to a pot weight at 5300 g.

In the first experiment, four drought treatments were applied (based on the SGWC, as described above): control—90% FC, IB40–40% FC at initial branching (IB), FL40–40% FC at flowering (FL) and IB50 + FL50—a combination of 50% FC at IB and 50% FC at FL. For IB40, drought stress was applied at the initial branching stage, by withholding water until the pot weight reached 4400 g (40% FC) and was maintained at this weight for two weeks by watering when necessary, then allowed to recover by gradually watering an increment rate of 150 ml per day to bring the pot weight to 5300 g (90% FC). For FL40, drought stress was imposed at the beginning of the flowering stage by withholding water, maintaining pot weight at 4400 g for two weeks, then allowed to recover by gradually watering at an increment of 150 ml per day and bringing the pot weight to 5300 g. For IB50+FL50, water was withheld at the initial branching stage until pots weigh reached to 4600 g (50% FC), which was maintained for two weeks; pots were allowed to gradually recover to 90% FC by watering with an increment of 150 ml per day. When plants reached flowering stage, water was once again withheld until pots reached 50% FC, held there for two weeks and gradually watered to bring the soil moisture at 90% FC. The plants under control treatment were constantly watered and maintained at 5300 g (90% FC) throughout the growth cycle. Pots were rotated daily through the automated weighing and watering stations, where each pot was weighed before and after dispensing of water, allowing maintenance of the required FC. The assembly of automated weighing and watering station is shown in the Fig 1B. Based on the results for shoot dry biomass and estimated green shoot biomass obtained from the first experiment, two drought levels, control and IB50 + FL50 were selected to screen diverse safflower genotypes.

### Image acquisition and processing

**Fig 2:**
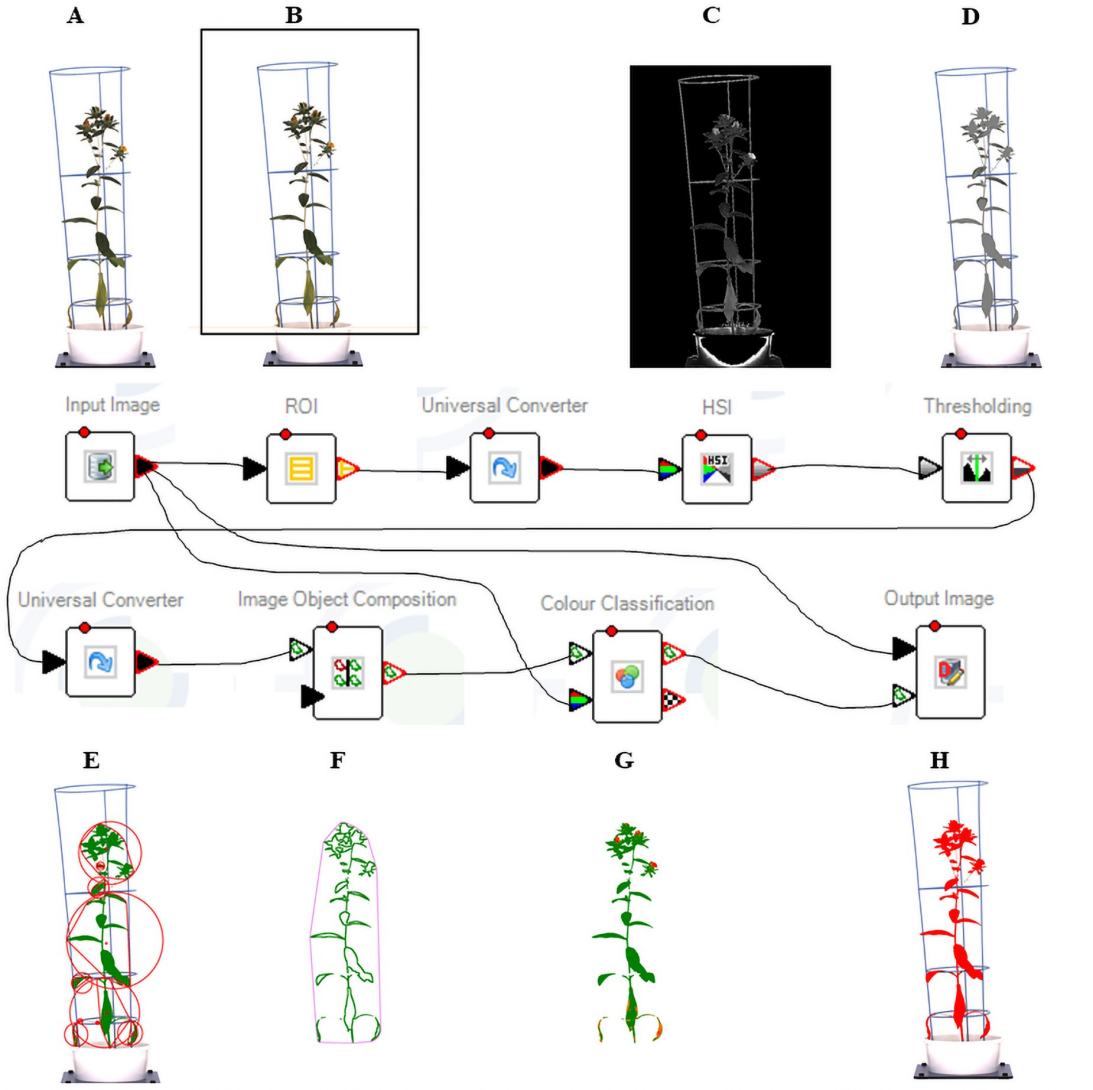
Simplified analysis pipeline for digital images. The principal steps and their transitional output images are displayed: (A) input image; (B) region of interest selection; (C) transformation of image to different colour scale such as HSI (hue, saturation intensity), (D) thresholding applied; (E) multiple individual objects identified; (F) merging of individual objects into one object as image object composition; (G) plant colour classification; and (H) detection of plant.

High-resolution digital images were acquired using top and side mounted RGB Prosilica GT 6600C cameras (Allied Vision Technologies, Stadtroda, Germany), capturing three side views (0°, 120° and 240°) and a top view (Fig 1C). The acquired images were stored in a database server, then images were analysed through an image analysis pipeline developed in LemnaGrid (Lemnatec, GmBH, Aachen, Germany). An overview of image analysis pipeline with intermediary results is provided in Fig 2. Initially the region of interest was defined on the raw image to eliminate pixels from imaging unit (cabinet edges, conveyor, turner and lifter). The resulting image was transformed into other colour spaces such as hue, saturation, intensity (HSI) and L*a*b (L*—lightness, a*—colour from green to magenta, b*—colour from blue to yellow) to improve the visibility of plant features. The logical operation was used to combine the results of HSI and L*a*b. An adaptive thresholding was applied on the images to eliminate the background, and further improve plant detection. A median filter was applied on the images to smoothen the edges, and the morphological operation such as erosion and fill areas, was applied to eliminate small, individual, unwanted pixels. Image object composition was used to combine spatially independent objects into one object, the plant. Colour classification, using nearest-neighbour method, was applied to identify green and non-green tissue. The estimated shoot biomass (ESB) was calculated by combining the pixel values from three side views and one top view. Other digital traits such as convex hull area (CHA), caliper length (CL) and minimum area rectangle (MAR) were estimated from processed images.

### Destructive harvesting and phenotypic measurement

In the first experiment, plant parameters including branch number (BrN), bud number (BdN) and plant height (PH) were manually counted or measured when the plants reached physiological maturity. Plants were harvested destructively at 186 DAS, separated into biomass and buds, with the biomass dried at 70°C for 3 days and the buds dried at 40°C for 5 days, then added together to record the total dry biomass (DB). The buds were hand threshed and seeds were cleaned to record seed yield (g) (SY) and seed number (SN). The details of traits measured and estimated are provided in the Table 1. In the second experiment, plants were destructively harvested at 146 DAS, and DB and SY observations were taken as above.

**Table 1:** Safflower traits estimated and measured by destructive and digital methods.

Trait	Description
Dry biomass (DB)	Whole plant above ground biomass measured at the time of physiological maturity (g)
Seed yield (SY)	Total seed yield obtained per plant (g)
Branches (B)	Manual count of total number of branches per plant
Plant height (PH)	Measured from the bottom of the plant to the tip (cm)
Seed number (SN)	Number of seeds obtained per plant
Estimated shoot biomass (ESB)	Number of pixels estimated from identified plant area combined from three side views and a top view (000 pixels)
Water loss (WL)	Measured by weighing pot before and after watering (g day^-1^)
Bounding box (BB)	The rectangular box enclosing the identified plant (pixels)
Convex hull area (CHA)	Area of the convex hull that encloses the identified plant (pixels)
Caliper length (CL)	Maximum distance from top of the plant to the bottom (pixels)
Minimum area rectangle (MAR)	Minimum rectangular area that encloses the identified plant (pixels)

### Statistical analyses

The first experiment was conducted using randomized complete block design with six replications. The second experiment was conducted as split-plot design with control and drought treatments as main plots and genotypes as sub-plots. Data analysis was performed using R software (https://cran.r-project.org). A linear regression with Pearsons’s correlation (r) was used to demonstrate the relationship between estimated and measured plant traits using the package “Psych”. The average ESB was calculated as best linear unbiased estimates (BLUEs), using “lme4” package in R. These BLUEs were plotted to obtain the dynamic growth rate of safflower genotypes. K-means cluster analysis was used for assessing the relationship between ESB and SY, and to classify the genotypes based on their performance in these growth and yield related traits [72]. The clustering was performed using “cluster” and visualised using “factoextra” packages in R. The primary drought indices such as YI [43], GMP [44], MP [45], TOL [45] and STI [44] were calculated (Table 2) to assess the performance of genotypes under stress conditions. Similar to YI, with minor modifications, secondary drought indices such as BrNI (BrN), BdNI (BdN), PHI (PH), DBI (DB), SNI (SN) and ESBI (ESB) were calculated for morphological and yield related traits from the first experiment (Table 2).

**Table 2:** The primary and secondary drought indices used in the safflower experiment conducted at PPV, Horsham.

Drought index	Formula	Reference
**Yield index (YI)**	$\frac{Ys}{Yp}$	[43]
**Geometric mean productivity (GMP)**	$\sqrt{Yp\;\times Ys}$	[46]
**Mean productivity (MP)**	$\frac{Yp\;+Ys}{2}$	[45]
**Tolerance index (TOL)**	*Yp* − *Ys*	[45]
**Stress tolerance index (STI)**	$\frac{Yp\;\times Ys}{{\left(\overset{-}{Yp}\right)}^{2}}$	[46]
**Branch number index (BrNI)**	$BrNI\;=\;1-\left(\frac{{\text{X}}_{t}}{{\text{X}}_{c\;\left(mean\right)}}\right)$	This manuscript
**Bud number index (BdNI)**	$BdNI\;=\;1-\left(\frac{{\text{X}}_{t}}{{\text{X}}_{c\;\left(mean\right)}}\right)$	This manuscript
**Plant height index (PHI)**	$PHNI\;=\;1-\left(\frac{{\text{X}}_{t}}{{\text{X}}_{c\;(mean)}}\right)$	This manuscript
**Dry biomass index (DBI)**	$DBNI\;=\;1-\left(\frac{{\text{X}}_{t}}{{\text{X}}_{c\;(mean)}}\right)$	This manuscript
**Seed number index (SNI)**	$SNI\;=\;1-\left(\frac{{\text{X}}_{t}}{{\text{X}}_{c\;(mean)}}\right)$	This manuscript
**Estimated shoot biomass index (ESBI)**	$ESBI\;=\;1-\left(\frac{{\text{X}}_{t}}{{\text{X}}_{c\;(mean)}}\right)$	This manuscript

Where *Yp* and *Ys* are yields at control and stress respectively; $\overset{-}{Yp}$ mean yields of all genotypes under control treatment; X_*t*_ is the mean value for the respective trait for all genotypes under a particular stress treatment, and X_*c* (*mean*)_ is the mean value for the respective trait for of all genotypes under control treatment.

## Results

### Analysis of safflower growth under defined drought stress treatments

**Fig 3:**
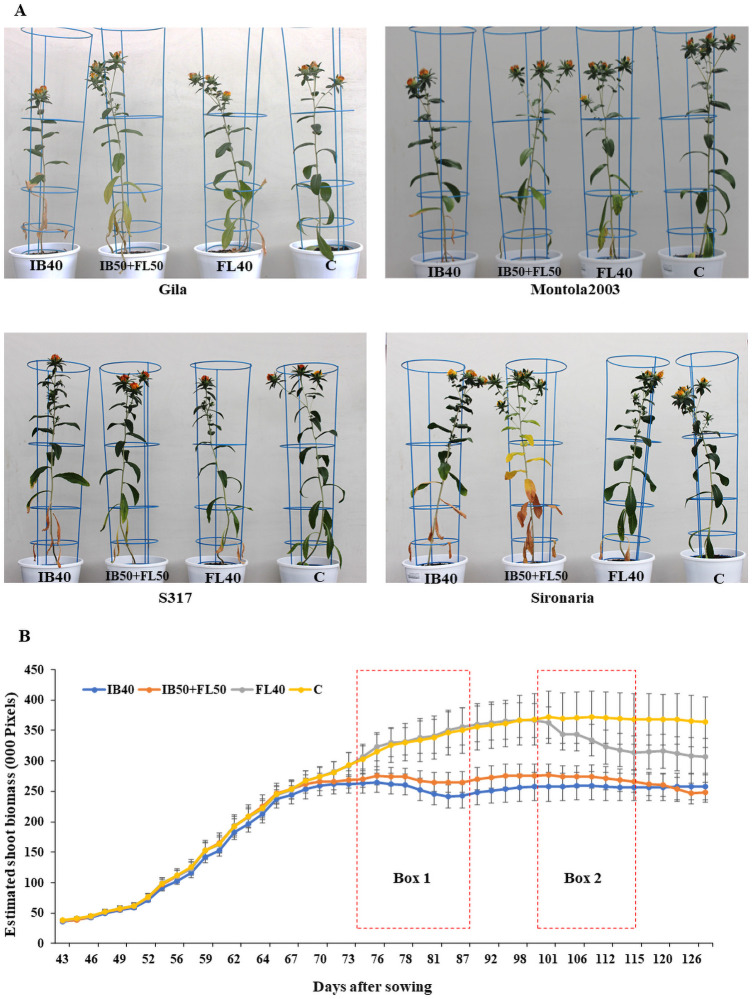
Growth of safflower plants under four different drought treatments. (A) The variations in growth of different safflower varieties under the four drought treatments, taken during flowering stage. (B) Estimation of shoot biomass and dynamic growth curves of safflower plants under different drought stress levels. Red dotted boxes Box 1 and Box 2 represent initial branching (IB) and flowering (FL) stages when drought stress treatments were imposed. C—control; IB40–40% field capacity (FC) at IB; F140–40% FC at FL; IB50+FL50–50% FC at both IB and FL. Data represent mean + SEM.

Four safflower genotypes were grown and assessed under four different watering treatments in the first experiment. Fig 3A illustrates the effects the different treatments had on growth at the flowering stage. Growth curves for each treatment were identical during initial growth stages until 66 DAS, when the majority of plants reached IB, and stress was imposed for two groups (Fig 3B). From here, the growth curves for two drought treatment (IB40 and IB50+FL50) separated, while the other two treatments were maintained at 90% FC (Fig 3B). The growth curve for FL40 separated from control after drought stress was imposed at FL stage (100 DAS). At harvest, clear differences in biomass production were seen between three groups: control, FL40 and IB40/IB50+FL50. In the second experiment, growth curves started to differ between control and IB50+FL50 plants, soon after stress was imposed at IB and differences were maintained throughout stress at FL until maturity (S2 Fig).

### Defining drought stress levels for safflower plants

**Fig 4:**
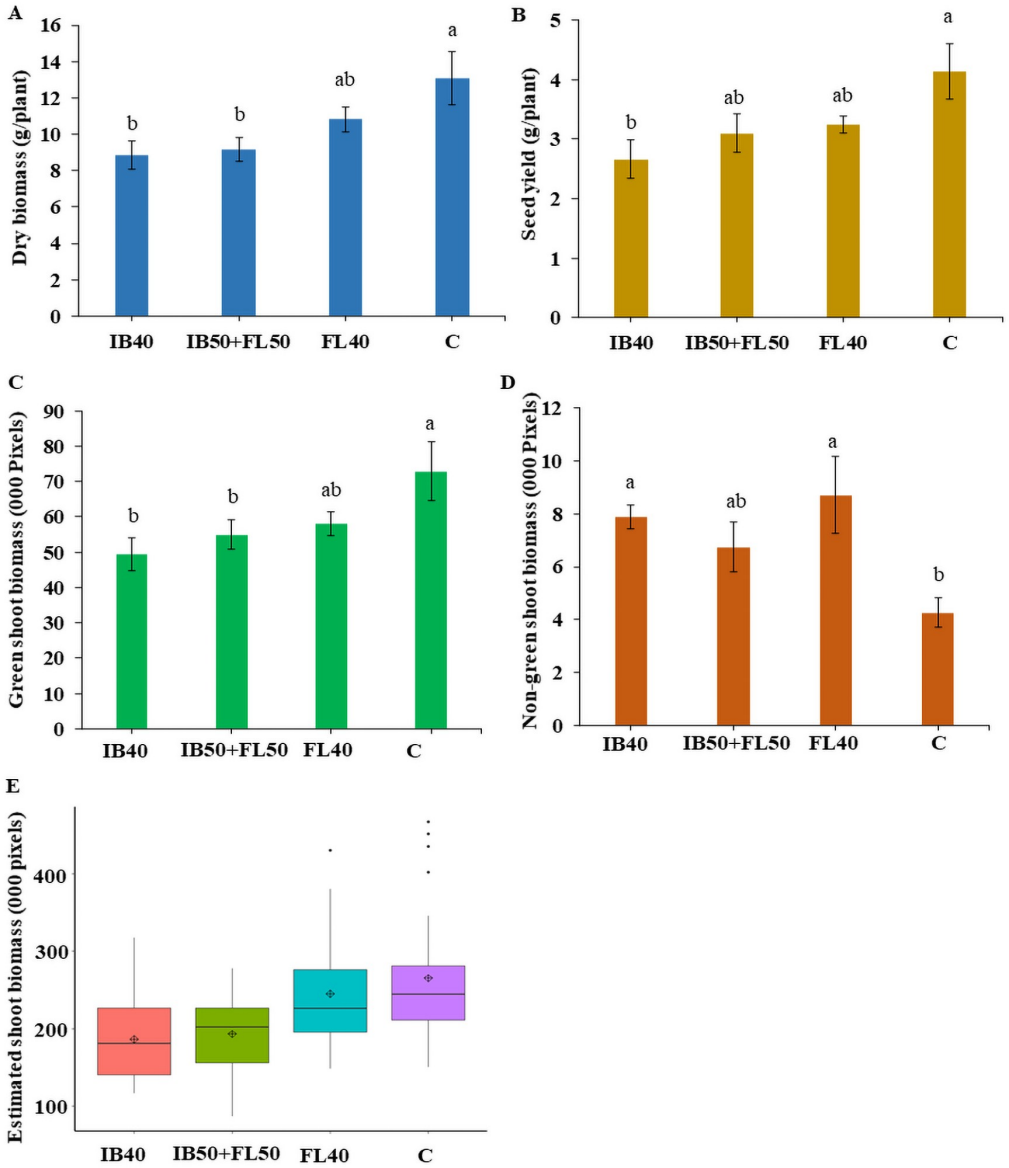
Performance of safflower genotypes under four different drought treatments. (A) Dry biomass for all four genotypes at the time of the maturity (g/plant). (B) Seed yield for all four genotypes at harvest (g/plant). (C) Estimated green shoot biomass and (D) estimated non-green shoot biomass from digital image analysis during flowering period. (E) Total estimated digital shoot biomass at end of imaging. C—control; TB40–40% field capacity (FC) at initial branching (IB); FL40–40% FC at flowering (FL); IB50+FL50–50% FC at both IB and FL. Each stress treatment was compared against the control. Data show mean + SEM. Different letters above each bar in the bar chart indicate significant differences between treatments (p < 0.05). The boxes in the boxplot represent lower and upper quartile, vertical lines attached to each bar is whisker. The black horizontal line indicates median; diamond plus represents mean.

In the first experiment, safflower plants were grown under four different watering treatments, to identify and assess the ideal drought stress level for shoot biomass and seed yield (Fig 4 and S1 Fig). As expected, plants produced increased DB and SY in treatments with higher availability of water, although due to variation between genotypes no significant differences were observed between the three drought treatments (Fig 4A and 4B). IB40 plants were the most susceptible to drought stress, producing the least DB and SY, as well as producing relatively low green biomass, with relatively high amounts of non-green tissue (Fig 4C and 4D). Meanwhile, FL40 plants performed relatively the best of the three stress treatments, producing relatively high amounts of biomass, although a considerable portion consisted of non-green tissue (Fig 4). IB50+FL50 plants had similar biomass to IB40 and similar seed yields to FL40 plants, although interestingly compared to other drought treatments, a relatively low proportion was non-green biomass. Control plants produced the most DB and SY, with the least proportion of non-green biomass (Fig 4). Based on the results of the first experiment, and taking into consideration the known drought sensitivity of safflower at IB and FL stages [15], treatment IB50+FL50 was chosen to screen diverse genotypes for water stress tolerance in the second experiment. IB50+FL50 was also selected as it would ensure a clear difference between control and water stressed plants, unlike FL40, but was not too severe like IB40, which may have skewed data collection from very sensitive genotypes.

### Estimation of digital plant traits

**Fig 5:**
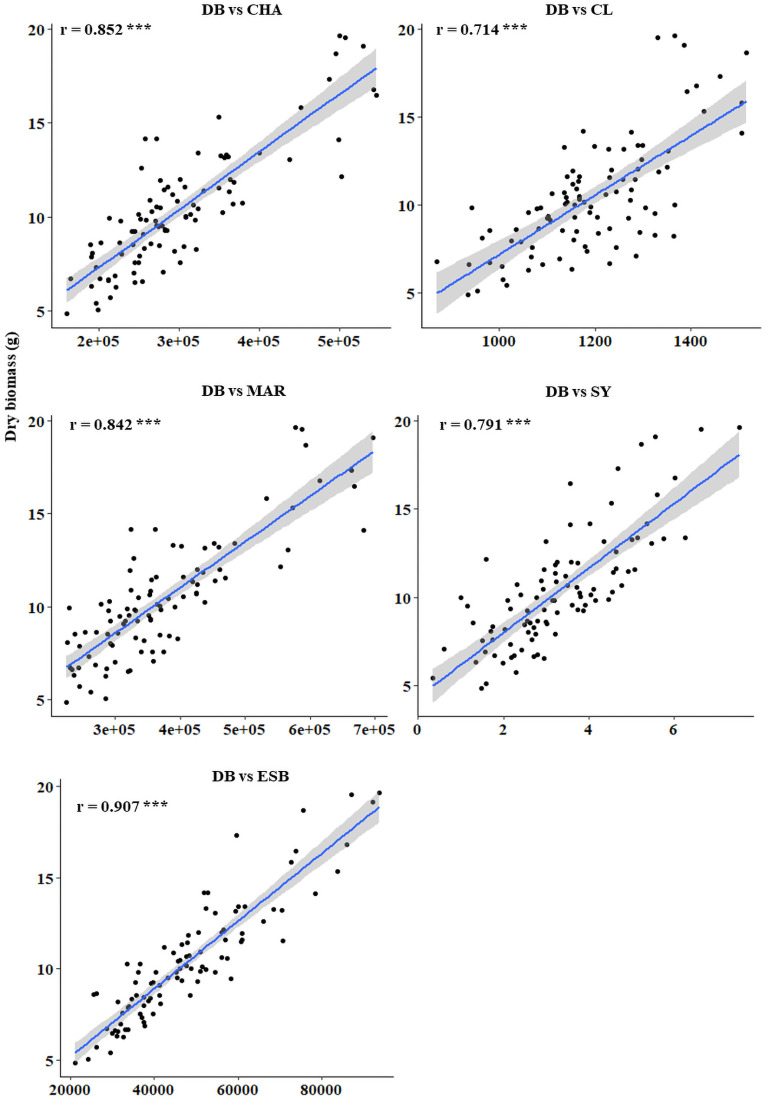
Correlations between dry biomass with other plant traits calculated from the screening of 200 diverse safflower genotypes. DB. dry biomass; CHA, convex hull area, CL. caliper length, MAR. minimum area rectangle; SY. seed yield; ESB. estimated shoot biomass; r, coefficient of correlation. Blue line indicates the line of best fit as a result of linear regression. The grey shaded area represents the confidence interval. The asterisks show the significance level (*** p< 0.001).

Comparing the patterns of growth across the four water stress treatments illustrated that trends in manual DB were mirrored in ESB (Fig 4A and 4E). The two traits were found to be significantly tightly correlated, having a correlation coefficient (r) of 0.91 (Fig 5) in the first experiment, and r = 0.88 in the second experiment (S3B Fig), suggesting the two traits can be interchanged. The DB also showed significantly high positive correlation with CL (r = 0.698) and PH (r = 0.679) (S3A Fig) indicating that the non-destructive CL measurement can be effectively used as surrogate measure for DB. Plants grown under IB40 produced relatively low green biomass, with relatively high amounts of non-green tissue; while plants under FL40 showed relatively high amounts of ESB, of which a considerable portion consisted of non-green tissue (Fig 4C and 4D). IB50+FL50 plants produced low ESB, but interestingly compared to other drought treatments, relatively low proportions of non-green biomass.

**Fig 6:**
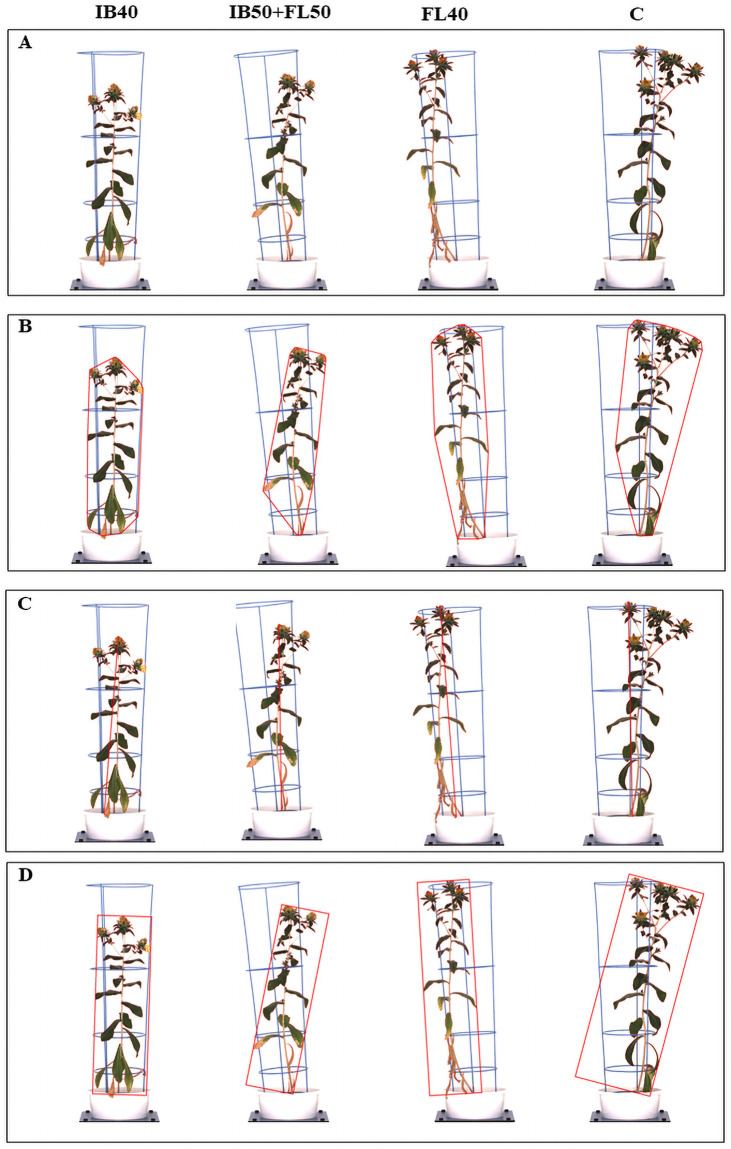
Estimation of plant digital traits. (A) Identification of colour classified whole plant; ESB, (B) convex hull area; CHA, (C) caliper length; CL. (D) minimum area rectangle; MRA. Each vertical panel represents plants grown at IB40–40% field capacity (FC) at initial branching (IB); FL40–40% FC at flowering (FL); IB50+FL50–50% FC at both IB and FL; and C—control.

The relationship between DB and other estimated plant traits was examined using Pearson’s correlations, which showed strong correlations between DB and CHA (r = 0.852), CL (r = 0.714), MAR (r = 0.842) and SY (r = 0.791) (Fig 5). Fig 6 visualises how digital plant traits CL (an estimate of plant height), CHA and MAR (which both indicate plant spread) were calculated, as well as responses to the different water stress treatments. In concurrence with biomass trends, control plants which produced more biomass showed higher CHA, CL and MAR compared to the plants under IB40 which had the least spread and height (Fig 6). The ESB and SY traits from second experiments, showed significantly high correlation (r = 0.619), which were used to cluster genotypes based on estimated and measured parameters (S4 Fig).

### Assessment of drought stress indices and comparative performance of safflower genotypes

**Fig 7:**
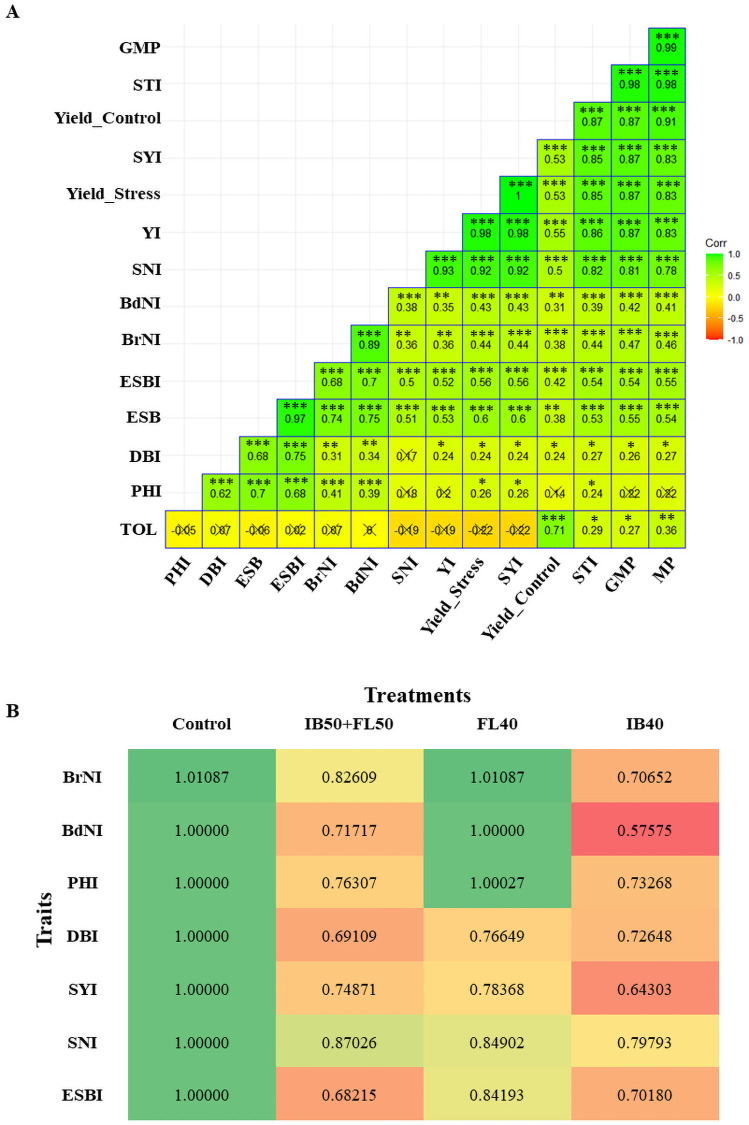
Performance of genotypes against stress related indices. (A) Correlation matrix and (B) heatmap of stress indices showing the performance of morphological and yield related traits under control and stress levels. In correlation matrix values are correlation coefficients (r), *** P < 0.001, ** P< 0.01, * P < 0.05, cross (x) on the r values indicate non significant, GMP, Geometric mean productivity. MP, mean productivity; STI, stress tolerance index; SYI. seed yield index; YI, yield index; SNI. seed number index; BdNIbud number index; BrNI, branch number index; ESBI, estimated shoot biomass index; ESB, estimated shoot biomass; DBI. dry biomass index, PHI, plant height index; TOL, tolerance index. In the heatmap, the green cell colour with higher index values indicates no effect or improvement under stress treatment, while orange to red cell colours with lower index values shows negative affects of stress treatment. C—control; IB40–40% field capacity (FC) at initial branching (IB); FL40–40% FC at flowering (FL); IB50+FLS0–50% FC at both IB and FL.

To determine how plant parameters reacted to varied stress levels, drought indices such as YI, GMP, MP, TOL and STI were calculated. Based on the YI formula, other indices such as BrNI, BdNI, PHI, DBI, SNI and ESBI were calculated. A correlation matrix showing the relationship of ESB with primary and secondary drought indices, yield at stress and control is presented in the Fig 7A. The ESB showed moderate positive correlation with drought indices YI (r = 0.53), STI (r = 0.53), GMP (r = 0.55) and MP (r = 0.54) (Fig 7A). Yield at stress and control treatments showed high correlations (r = 0.87) with GMP index. Values below one for respective plant parameters under a given treatment indicated that these traits were negatively affected by stress, while index values at or above one indicated the trait was not affected (Fig 7B). Plants grown under control conditions, showed better performance in all traits than the plants screened under all stress treatments. Drought treatment FL40 only negatively affected traits determined post water stress. SN and SY, as expected, showed reduced index values under drought stress, which impacted on the final DB (Fig 7). All measured and estimated parameters were negatively affected under IB40 and IB50+FL50 treatments, especially under IB40 treatment, which reflected the reduced performance of plants. Using the established protocol, 200 safflower genotypes were assessed in the second experiment, for yield stability to determine drought tolerance. The association between ESB and SY showed a moderately high positive correlation (r = 0.619) (S3B Fig) in plants under drought stress treatment. This association was used to assess and group the performance of genotypes under drought stress treatment IB50+FL50, using cluster analysis. Analysis showed four clusters formed: Cluster 1 –high biomass with high yield; Cluster 2 –medium biomass with medium to high yield; Cluster 3 –low biomass with low yield; Cluster 4 –very low biomass with very low yield (S4 Fig).

## Discussion

The present research describes the use of precise, reliable and high-throughput image-based phenotyping to screen large numbers of safflower genotypes, based on the evaluation of varying levels of defined drought stress under controlled environments. Although safflower is relatively drought tolerant, acute water scarcity can have significant negative effects on growth and development. For example, early and untimely leaf senescence can result in inadequate remobilization of stored metabolites from older to growing tissues, affecting yield and quality [73]. The ability to select genotypes with increased tolerance to water stress is paramount, as recent climate change models have forecast a substantial increase in global temperatures and drought conditions, resulting in a scarcity of water for growing of crops [74].

The study of drought incidence and progression under natural field situations is complex due to the involvement of many heterogeneous variables [75]. Controlled environment drought studies facilitate in removing some of these variables [76], which will aid in the selection of safflower genotypes based on the performance of yield and related traits. Traditional phenotyping methods to select for drought tolerance are often time and cost ineffective, and less reliable due to single timepoint measurements. New generation high-throughput phenotyping technologies play a critical role in hastening the selection of elite genotypes that are highly water efficient [77, 78]. Image-based measurements form a key component of current high-throughput phenotyping technology, where pixel values can be used as surrogates to measure biomass and other plant parameters [79].

Two independent experiments were conducted to assess the reliability and repeatability of using digital image-based phenotyping to assess drought tolerance in safflower. The results illustrated that safflower clearly responded to the availability of water, evident as plants under treatments IB40 and IB50+FL50 recorded the lowest DB, ESB and SY, while control plants had the highest. Other studies have reported similar results, with reductions in yield and harvest indices observed in other safflower genotypes exposed to drought stress during either vegetative and flowering stages [22, 26, 29, 31, 40, 41, 80]. Reductions in fresh and dry weights associated with increased severity of water stress have been observed in other crops including sunflower [81] and wheat [82]. In the first experiment, other parameters evaluated showed that vegetative (BrN, BdN) and reproductive (SY, SN) traits were also depressed in plants grown under IB40 and IB50+FL50, in contrast to control. Indicating that the plants under these two treatments experienced significantly higher stress due to the low availability of water at critical growth stages. Vegetative growth around the branching stage has previously been shown to be highly sensitive to drought, with water stress not only affecting biomass production but also yield parameters [22, 26, 40, 80]. FL40 treated plants were only affected negatively by stress in DB and YI values, indicating that water stress at critical bud formation, flowering and grain filling stages can significantly reduce seed yield and quality, but has little effect on other growth parameters, consistent with previous literature [22, 29, 31, 41, 80].

Drought indices are key tools for assessing genetic differences between genotypes relating to water stress tolerance. Indices such as stress tolerance (STI) [46], yield (YI) [43], stress susceptibility (SSI) [83], tolerance (TOL) [45] and yield stability indices (YSI) [84] have been previously studied by deriving mathematical relationships between yield under stress and control conditions. Genotypes that exhibit drought tolerance have higher values of YI (59), GMP [44], MP [45] and STI [44] and play a pivotal role in the selection of drought tolerant genotypes. Similar trends were seen for the vegetative and reproductive index values investigated in the first experiment. Morphological indices play critical roles in early selection of the drought tolerant genotypes [32], and improve the accuracy of selection in safflower breeding programs.

Image-based measurements were acquired at regular timepoints during the safflower growing period. These precise, image-based measurements were used to establish dynamic growth curves across multiple timepoint, over the four different treatments. Growth curves for IB40 and IB50+FL50 treatments segregated from control treatment at IB, indicating a quick response to water stress. Both these treatments had decrease in plant growth, and yield parameters, even IB40 which was resupplied with water during FL and grain filling. This indicates that drought at IB affects plant biomass production by reducing the BrN and therefore limit the number of buds able to be produced, which can have a more severe impact on final yield, than stress which only affects seed formation. Meanwhile, the FL40 growth curve showed that biomass was not lost until initiation of drought stress at FL, which was likely due in part to undevelopment of buds, which resulted in reduced SN and SY. Based on the analysis of results from the first experiment, control and IB50+FL50 stress treatments were chosen to screen 200 diverse safflower genotypes.

In the second experiment, growth curves for the two treatments were similar until the initiation of stress at IB, where curves separated from each other and continued to segregate during FL. Destructive harvesting at the end of the growing cycle validated the differential growth among safflower genotypes seen in the digital phenotyping evident by the variation in DB and SY. The strong association between biomass and yield has been well documented in the previous studies [42, 85, 86]. Strong positive correlations between ESB and SY and other drought indices such as STI, GMP and MP (Fig 7A) emphasise the role biomass can play in selecting desired drought tolerant genotypes at earlier growth stages in crops [56, 87]. Therefore, ESB from digital phenotyping could be used to effectively decipher drought responses between safflower genotypes, without the need for manual and destructive observations.

The relationship between biomass and SY has been previously used to classify genotypes into different clusters [88]. The ESB and SY obtained from the second experiment were used to perform the cluster analysis which grouped the genotypes into four clusters based on their responses to drought stress. Cluster 1 comprised of genotypes which produced high biomass and high seed yields, indicating that these genotypes were relatively drought tolerant and able to maintain growth and yield. The genotypes that fall under cluster 1 are good candidates to include in the safflower breeding program for introgression of drought tolerance trait. Cluster 2 included safflower genotypes with moderate biomass and yield, demonstrating moderate levels of drought stress tolerance. Cluster 3 encompassed genotypes which produced lower biomass and yield, signifying that genotypes in this cluster experienced relatively higher drought stress and were moderately drought susceptible. Cluster 4 represented genotypes which produced the lowest biomass and seed yield, and therefore were the most drought susceptible. Furthermore, the grouping of genotypes in cluster analysis, reiterated the tight correlation between the ESB and SY in safflower.

Digital RGB imaging technologies used in plant phenotyping have provided a range of opportunities to study morphological markers in crops such as maize [89], field pea [56], wheat [54, 55] and rice [90, 91] under different growing environments. Digital estimated parameters CHA and CL, which represent the smallest polygon area that covers the plant and plant height, respectively, can be used to provide details on the extent of plant spread, which can be interpreted by DB and BrN in safflower. Higher values for both parameters often indicates higher plant biomass [54], shown by strong correlations between all estimated plant biomarkers including CHA, CL and ESB with measured DB across all drought levels. Strong positive correlations between estimated and measured biomass have also been observed in barley [64] and wheat [53, 54]. The association of DB with PH and CL improves the accuracy in selecting the drought tolerant genotypes in the breeding programs.

Colour classification, which forms an important part of digital image analysis, is critical in identifying green and non-green (chlorotic/necrotic) tissues. The nearest-neighbour method, a simple algorithm, was employed to classify leaf tissues based on colour into green and non-green tissues. The image analysis pipeline used in this research efficiently classified green and non-green tissue, showing that control plants had significantly low numbers of non-green pixels compared to stress treatments. The efficient use of this algorithm has been demonstrated in identification of green and non-green leaf tissues under abiotic stresses including salinity in rice [91] and nitrogen stress in wheat [92], as well as identifying inter and intra plant structural variations [93]. These results demonstrate that RGB imaging can be effectively used to digitally quantify safflower traits in a non-destructive manner, contributing to the precise and rapid selection of genotypes in drought tolerance breeding. As these are non-destructive digital image-based measurements acquired at regular intervals, it is possible to track a more realistic dynamic growth of any crop under investigation.

In conclusion, non-invasive, repetitive, high-throughput, proximal sensing technology can be used to estimate the growth of safflower under drought stress conditions. The results showed that estimated and measured traits had strong correlations, suggesting the feasibility of using digital traits, in particular ESB, CHA, CL and MAR, and drought stress indices, as surrogates to destructive measurements, to improve the accuracy and reliability of selections in crop breeding programmes. Future work in this area is needed to investigate the responses of candidate genotypes, identified using digital phenotyping, under a field-based environment where natural drought occurs at varied intensity and growth stages.

## Supporting information

S1 Fig Weights of pots undergoing four different drought stress treatments. The green dashed line is control, red dashed line 40% field capacity (FC) at initial branching (IB) (IB40), blue dashed line 40% FC at flowering (FL) (FL40) and orange dashed line 50% FC at both IB and FL (IB50+FL50). Data represent mean ± SEM.
(TIF)

S2 Fig Dynamic growth of safflower plants under control and drought (IB50+FL50) stress. Boxplots show the changes in estimated shoot biomass over the duration of the experiment, illustrating the variation in growth patterns for the 200 diverse safflower genotypes screened. Boxplots show the lower and upper quartiles with the black horizontal lines in the box represent median; the vertical lines attached to the box indicate whiskers; the black dots show the extreme data points. Shoot biomass at control (green boxes) and drought stress (red boxes). The red dashed boxes represents the periods where drought stress was imposed during initial branching and flowering stages.
(TIF)

S3 Fig Correlations between dry biomass with other plant traits calculated from the screening of diverse safflower genotypes in (a) experiment 1 and (b) experiment 2. DB, dry biomass; CL, caliper length; PH, plant height; ESB, estimated shoot biomass; SY, seed yield; r, coefficient of correlation. Blue line indicates the line of best fit as a result of linear regression. The grey shaded area represents the confidence interval. The asterisks show the significance level (*** p < 0.001).
(TIF)

S4 Fig Cluster analysis between estimated shoot biomass and seed yield. Four clusters formed to demonstrate the relationship between the measured plant traits.
(TIF)

S1 Table Diverse safflower genotypes sourced from the Agriculture Victoria safflower breeding population utilised in the controlled environment experiments. (DOCX)
