# Supplementary figures and images for: High-throughput phenotyping to dissect genotypic differences in safflower for drought tolerance
Source: PLoS One. 2021 Jul 23;16(7):e0254908. doi: 10.1371/journal.pone.0254908 (PMC8301646; doi:10.1371/journal.pone.0254908)

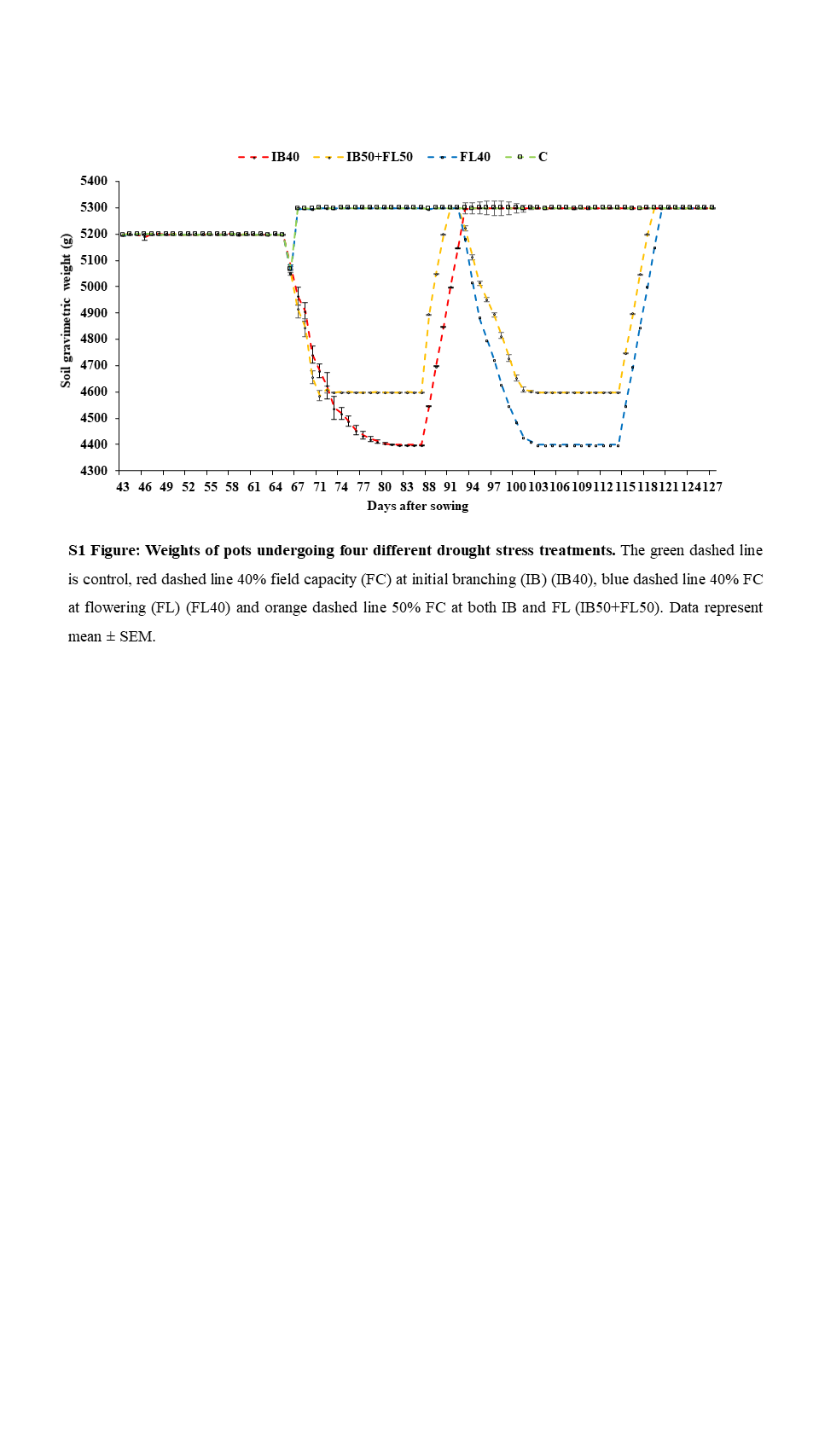

Supplement: S1 Fig — The green dashed line is control, red dashed line 40% field capacity (FC) at initial branching (IB) (IB40), blue dashed line 40% FC at flowering (FL) (FL40) and orange dashed line 50% FC at both IB and FL (IB50+FL50). Data represent mean ± SEM. (TIF) [file pone.0254908.s001.tif]

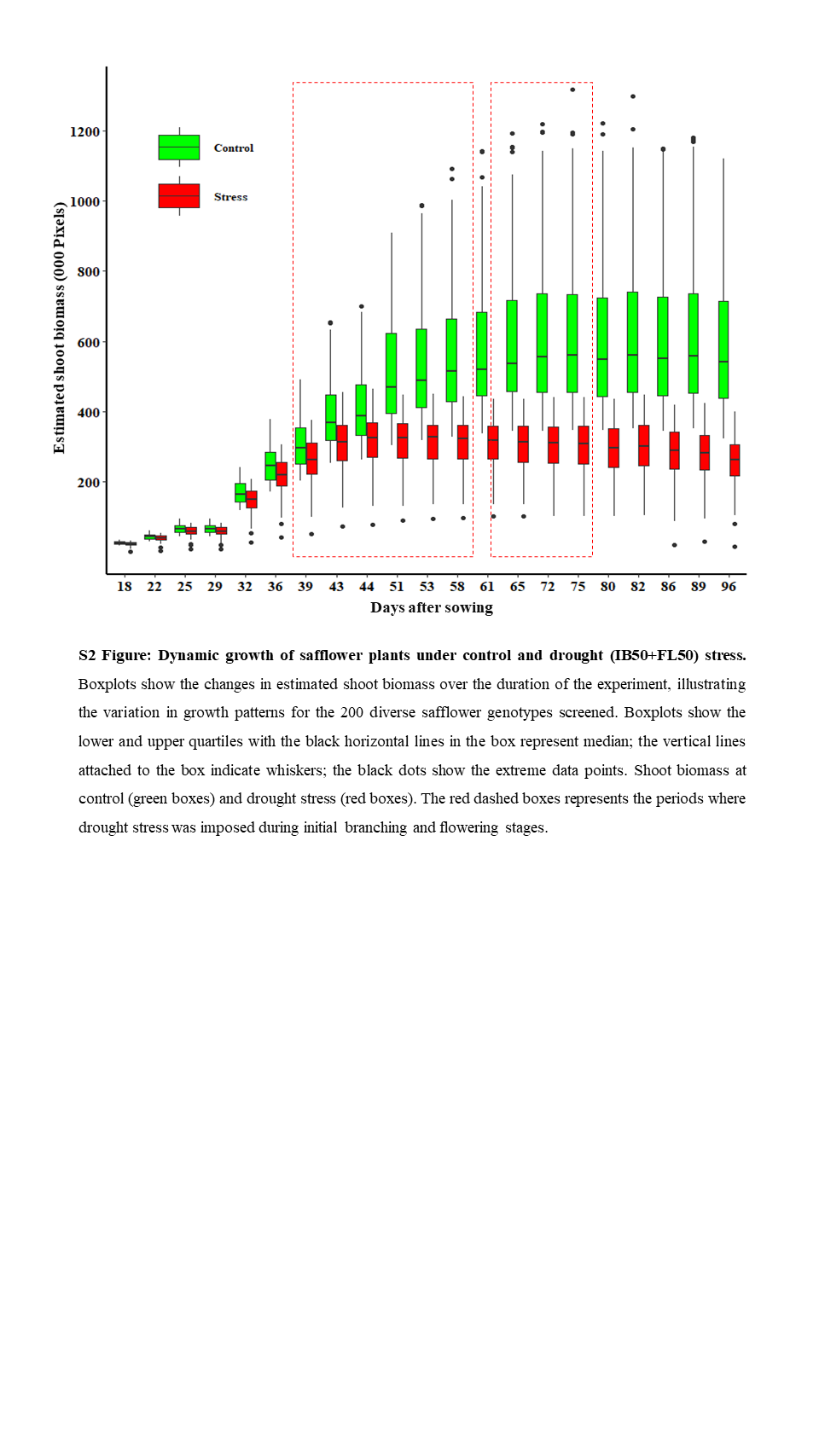

Supplement: S2 Fig — Boxplots show the changes in estimated shoot biomass over the duration of the experiment, illustrating the variation in growth patterns for the 200 diverse safflower genotypes screened. Boxplots show the lower and upper quartiles with the black horizontal lines in the box represent median; the vertical lines attached to the box indicate whiskers; the black dots show the extreme data points. Shoot biomass at control (green boxes) and drought stress (red boxes). The red dashed boxes represents the periods where drought stress was imposed during initial branching and flowering stages. (TIF) [file pone.0254908.s002.tif]

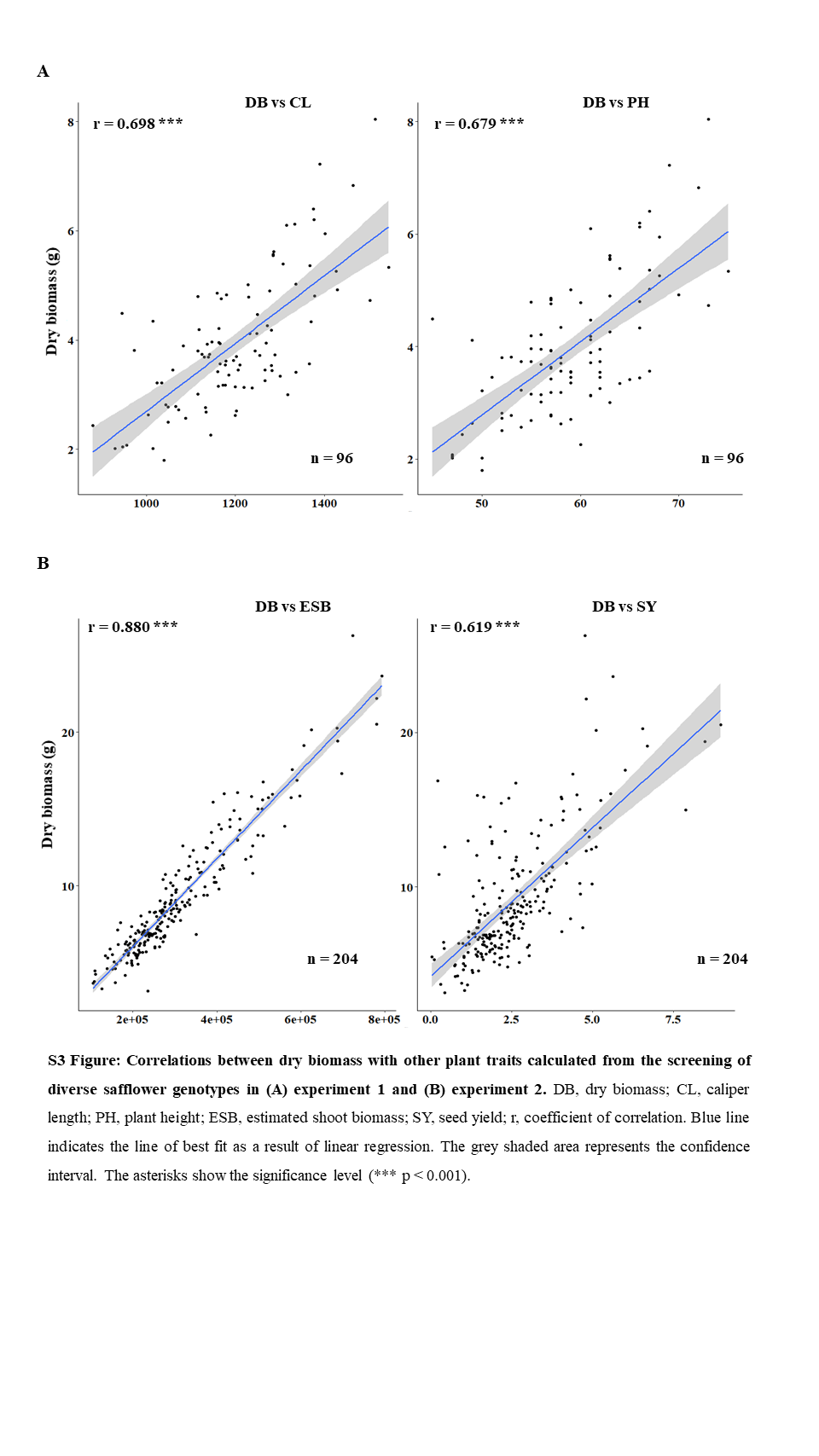

Supplement: S3 Fig — Correlations between dry biomass with other plant traits calculated from the screening of diverse safflower genotypes in (a) experiment 1 and (b) experiment 2. DB, dry biomass; CL, caliper length; PH, plant height; ESB, estimated shoot biomass; SY, seed yield; r, coefficient of correlation. Blue line indicates the line of best fit as a result of linear regression. The grey shaded area represents the confidence interval. The asterisks show the significance level (*** p < 0.001). (TIF) [file pone.0254908.s003.tif]

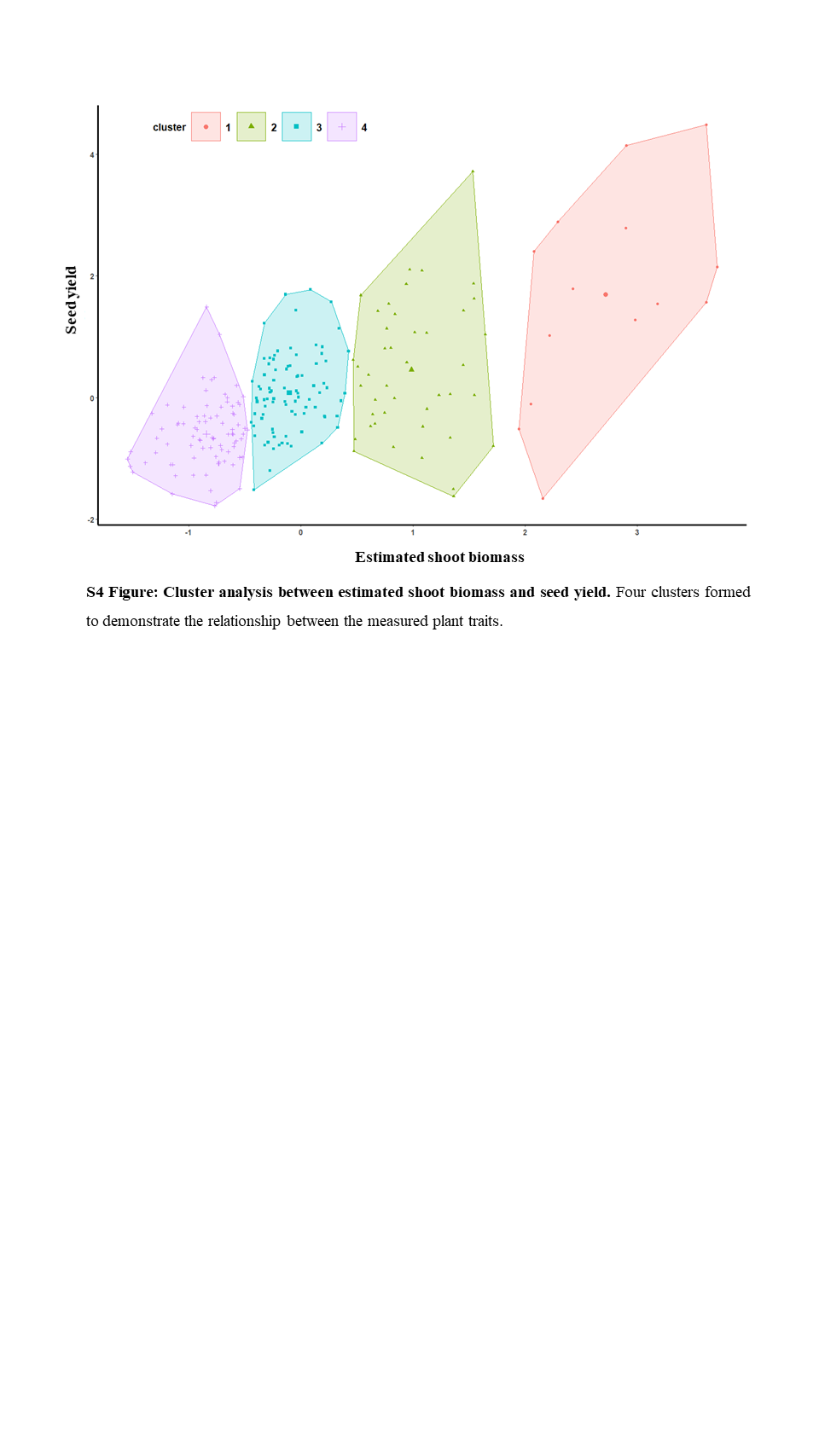

Supplement: S4 Fig — Four clusters formed to demonstrate the relationship between the measured plant traits. (TIF) [file pone.0254908.s004.tif]
