# Supplementary material for: High-throughput phenotyping to dissect genotypic differences in safflower for drought tolerance
Source: PLoS One. 2021 Jul 23;16(7):e0254908. doi: 10.1371/journal.pone.0254908 (PMC8301646; doi:10.1371/journal.pone.0254908)
Supplement: S1 Table — (DOCX) [file pone.0254908.s005.docx]

S1 Table: Diverse safflower genotypes sourced from the Agriculture Victoria safflower breeding population utilised in the controlled environment experiments.

| Genotype | Status | Country | Genotype | Status | Country |
| --- | --- | --- | --- | --- | --- |
| AVS-SAFF-4 | Breeder’s Line | Ethiopia | AVS-SAFF-80 | Breeder’s Line | India |
| AVS-SAFF-7 | Breeder’s Line | Israel | AVS-SAFF-83 | Breeder’s Line | India |
| AVS-SAFF-8 | Breeder’s Line | Sudan | AVS-SAFF-85 | Breeder’s Line | India |
| AVS-SAFF-11 | Breeder’s Line | Iran | AVS-SAFF-86 | Breeder’s Line | India |
| AVS-SAFF-12 | Breeder’s Line | Japan | AVS-SAFF-87 | Breeder’s Line | India |
| AVS-SAFF-13 | Breeder’s Line | Afghanistan | AVS-SAFF-88 | Breeder’s Line | India |
| AVS-SAFF-14 | Breeder’s Line | China | AVS-SAFF-89 | Breeder’s Line | India |
| AVS-SAFF-16 | Breeder’s Line | Unknown | AVS-SAFF-91 | Breeder’s Line | India |
| AVS-SAFF-17 | Breeder’s Line | Unknown | AVS-SAFF-92 | Breeder’s Line | India |
| AVS-SAFF-18 | Breeder’s Line | Unknown | AVS-SAFF-94 | Breeder’s Line | Unknown |
| AVS-SAFF-19 | Breeder’s Line | Unknown | AVS-SAFF-96 | Breeder’s Line | Unknown |
| AVS-SAFF-21 | Breeder’s Line | Unknown | AVS-SAFF-97 | Breeder’s Line | Unknown |
| AVS-SAFF-23 | Breeder’s Line | Unknown | AVS-SAFF-98 | Breeder’s Line | Unknown |
| AVS-SAFF-25 | Breeder’s Line | Unknown | AVS-SAFF-99 | Breeder’s Line | Unknown |
| AVS-SAFF-26 | Breeder’s Line | Unknown | AVS-SAFF-100 | Breeder’s Line | Unknown |
| AVS-SAFF-51 | Breeder’s Line | Unknown | AVS-SAFF-101 | Breeder’s Line | Unknown |
| AVS-SAFF-55 | Breeder’s Line | Unknown | AVS-SAFF-105 | Breeder’s Line | United States |
| AVS-SAFF-56 | Breeder’s Line | Unknown | AVS-SAFF-112 | Breeder’s Line | Turkey |
| AVS-SAFF-57 | Breeder’s Line | Unknown | AVS-SAFF-114 | Breeder’s Line | Sudan |
| AVS-SAFF-58 | Breeder’s Line | Unknown | AVS-SAFF-115 | Breeder’s Line | Egypt |
| AVS-SAFF-62 | Breeder’s Line | Unknown | AVS-SAFF-116 | Breeder’s Line | Egypt |
| AVS-SAFF-63 | Breeder’s Line | Former Soviet Union | AVS-SAFF-117 | Breeder’s Line | Turkey |
| AVS-SAFF-66 | Breeder’s Line | Kenya | AVS-SAFF-118 | Breeder’s Line | Turkey |
| AVS-SAFF-67 | Breeder’s Line | India | AVS-SAFF-121 | Breeder’s Line | Iran |
| AVS-SAFF-69 | Breeder’s Line | India | AVS-SAFF-124 | Breeder’s Line | Iran |
| AVS-SAFF-75 | Breeder’s Line | India | AVS-SAFF-129 | Breeder’s Line | Israel |
| AVS-SAFF-77 | Breeder’s Line | India | AVS-SAFF-130 | Breeder’s Line | United States |
| AVS-SAFF-78 | Breeder’s Line | India | AVS-SAFF-131 | Breeder’s Line | Unknown |
| Genotype | **Status** | **Country** | **Genotype** | **Status** | **Country** |
| AVS-SAFF-138 | Breeder’s Line | Unknown | AVS-SAFF-192 | Breeder’s Line | Kenya |
| AVS-SAFF-139 | Breeder’s Line | Unknown | AVS-SAFF-193 | Breeder’s Line | Kenya |
| AVS-SAFF-140 | Breeder’s Line | Unknown | AVS-SAFF-195 | Breeder’s Line | Portugal |
| AVS-SAFF-144 | Breeder’s Line | Unknown | AVS-SAFF-202 | Breeder’s Line | United States |
| AVS-SAFF-145 | Breeder’s Line | Unknown | AVS-SAFF-203 | Breeder’s Line | Kenya |
| AVS-SAFF-146 | Breeder’s Line | Unknown | AVS-SAFF-204 | Breeder’s Line | Pakistan |
| AVS-SAFF-148 | Breeder’s Line | Unknown | AVS-SAFF-206 | Breeder’s Line | Pakistan |
| AVS-SAFF-150 | Breeder’s Line | Unknown | AVS-SAFF-208 | Breeder’s Line | Pakistan |
| AVS-SAFF-151 | Breeder’s Line | Unknown | AVS-SAFF-209 | Breeder’s Line | Pakistan |
| AVS-SAFF-152 | Breeder’s Line | Unknown | AVS-SAFF-210 | Breeder’s Line | Pakistan |
| AVS-SAFF-162 | Landrace | Unknown | AVS-SAFF-213 | Breeder’s Line | Pakistan |
| AVS-SAFF-163 | Breeder’s Line | Ethiopia | AVS-SAFF-214 | Breeder’s Line | Pakistan |
| AVS-SAFF-164 | Breeder’s Line | Ethiopia | AVS-SAFF-216 | Breeder’s Line | Pakistan |
| AVS-SAFF-166 | Breeder’s Line | France | AVS-SAFF-218 | Breeder’s Line | Pakistan |
| AVS-SAFF-169 | Breeder’s Line | Algeria | AVS-SAFF-219 | Breeder’s Line | Pakistan |
| AVS-SAFF-170 | Breeder’s Line | Jordan | AVS-SAFF-220 | Breeder’s Line | Pakistan |
| AVS-SAFF-172 | Breeder’s Line | Afghanistan | AVS-SAFF-222 | Breeder’s Line | Pakistan |
| AVS-SAFF-173 | Breeder’s Line | Pakistan | AVS-SAFF-224 | Breeder’s Line | Pakistan |
| AVS-SAFF-174 | Breeder’s Line | Former Soviet Union | AVS-SAFF-225 | Breeder’s Line | Pakistan |
| AVS-SAFF-175 | Breeder’s Line | Ethiopia | AVS-SAFF-227 | Breeder’s Line | Pakistan |
| AVS-SAFF-177 | Breeder’s Line | Japan | AVS-SAFF-228 | Breeder’s Line | Pakistan |
| AVS-SAFF-178 | Breeder’s Line | Ethiopia | AVS-SAFF-230 | Breeder’s Line | Jordan |
| AVS-SAFF-179 | Breeder’s Line | Japan | AVS-SAFF-231 | Breeder’s Line | Israel |
| AVS-SAFF-181 | Breeder’s Line | Morocco | AVS-SAFF-232 | Breeder’s Line | Jordan |
| AVS-SAFF-183 | Breeder’s Line | Morocco | AVS-SAFF-233 | Breeder’s Line | Turkey |
| AVS-SAFF-184 | Breeder’s Line | Portugal | AVS-SAFF-234 | Breeder’s Line | Israel |
| AVS-SAFF-187 | Breeder’s Line | Turkey | AVS-SAFF-235 | Breeder’s Line | Syria |
| AVS-SAFF-190 | Breeder’s Line | Spain | AVS-SAFF-236 | Breeder’s Line | Portugal |
| AVS-SAFF-79 | Breeder’s Line | India | AVS-SAFF-237 | Breeder’s Line | Portugal |

| Genotype | Status | Country | Genotype | Status | Country |
| --- | --- | --- | --- | --- | --- |
| Montola2003 | Advanced cultivar | USA | AVS-SAFF-271 | Breeder’s Line | Turkey |
| PI 538025 | Advanced cultivar | USA | AVS-SAFF-277 | Breeder’s Line | Afghanistan |
| AVS-SAFF-237 | Breeder’s Line | Portugal | AVS-SAFF-278 | Breeder’s Line | Spain |
| AVS-SAFF-136 | Breeder’s Line | Unknown | AVS-SAFF-279 | Breeder’s Line | Former Soviet Union |
| AVS-SAFF-239 | Breeder’s Line | Portugal | AVS-SAFF-280 | Breeder’s Line | China |
| AVS-SAFF-241 | Breeder’s Line | Portugal | SIGMA 13 | Advanced cultivar | Unknown |
| AVS-SAFF-242 | Breeder’s Line | Portugal | SIGMA 16 | Advanced cultivar | Unknown |
| AVS-SAFF-243 | Breeder’s Line | Portugal | AVS-SAFF-191 | Breeder’s Line | Kenya |
| AVS-SAFF-244 | Breeder’s Line | Portugal | AVS-SAFF-281 | Breeder’s Line | Sudan |
| AVS-SAFF-245 | Breeder’s Line | Portugal | AVS-SAFF-284 | Breeder’s Line | Japan |
| AVS-SAFF-246 | Breeder’s Line | Israel | AVS-SAFF-306 | Breeder’s Line | India |
| AVS-SAFF-247 | Breeder’s Line | Pakistan | AVS-SAFF-307 | Breeder’s Line | India |
| AVS-SAFF-248 | Breeder’s Line | Argentina | AVS-SAFF-332 | Breeder’s Line | United States |
| AVS-SAFF-249 | Breeder’s Line | Israel | AVS-SAFF-333 | Breeder’s Line | United States |
| AVS-SAFF-250 | Breeder’s Line | Israel | AVS-SAFF-334 | Breeder’s Line | United States |
| AVS-SAFF-251 | Breeder’s Line | Pakistan | AVS-SAFF-335 | Breeder’s Line | United States |
| AVS-SAFF-252 | Breeder’s Line | Pakistan | AVS-SAFF-338 | Breeder’s Line | Unknown |
| AVS-SAFF-254 | Breeder’s Line | Iran | AVS-SAFF-341 | Breeder’s Line | Unknown |
| AVS-SAFF-257 | Breeder’s Line | Iran | AVS-SAFF-342 | Breeder’s Line | Unknown |
| AVS-SAFF-259 | Breeder’s Line | Iran | AVS-SAFF-343 | Breeder’s Line | Unknown |
| AVS-SAFF-260 | Breeder’s Line | Iran | AVS-SAFF-345 | Breeder’s Line | Unknown |
| AVS-SAFF-261 | Breeder’s Line | Iran | AVS-SAFF-347 | Breeder’s Line | Unknown |
| AVS-SAFF-262 | Breeder’s Line | Iran | AVS-SAFF-348 | Breeder’s Line | Unknown |
| AVS-SAFF-263 | Breeder’s Line | Turkey | AVS-SAFF-349 | Breeder’s Line | Unknown |
| AVS-SAFF-264 | Breeder’s Line | Turkey | AVS-SAFF-350 | Breeder’s Line | Unknown |
| AVS-SAFF-265 | Breeder’s Line | Turkey | AVS-SAFF-351 | Breeder’s Line | Unknown |
| AVS-SAFF-267 | Breeder’s Line | Turkey | AVS-SAFF-352 | Breeder’s Line | Unknown |
| AVS-SAFF-269 | Breeder’s Line | Turkey | AVS-SAFF-353 | Breeder’s Line | Unknown |

| Genotype | Status | Country | Genotype | Status | Country |
| --- | --- | --- | --- | --- | --- |
| AVS-SAFF-354 | Breeder’s Line | Unknown | SIGMA 45 | Advanced cultivar | Unknown |
| AVS-SAFF-355 | Breeder’s Line | Unknown | SIGMA 48 | Advanced cultivar | Unknown |
| AVS-SAFF-356 | Breeder’s Line | Unknown | SIGMA 50 | Advanced cultivar | Unknown |
| AVS-SAFF-361 | Breeder’s Line | Unknown | SIRONARIA | Advanced cultivar | Australia |
| AVS-SAFF-365 | Breeder’s Line | Unknown | UC 148 | Advanced cultivar | USA |
| AVS-SAFF-377 | Breeder’s Line | Bangladesh | USB | Advanced cultivar | USA |
| AVS-SAFF-379 | Breeder’s Line | Mexico | VARIETY 1 CSIRO ORD RIVER | Advanced cultivar | Unknown |
| BRIGGS | Advanced cultivar | Unknown | VARIETY 5 CSIRO ORD RIVER | Advanced cultivar | Unknown |
| cv.Hamaya 65 | Advanced cultivar | Unknown |  |  |  |
| cv.Kino 76 | Advanced cultivar | Unknown |  |  |  |
| SIGMA 46 | Advanced cultivar | Unknown |  |  |  |
| ROYAL | Advanced cultivar | USA |  |  |  |
| S317 | Advanced cultivar | USA |  |  |  |
| CW 99-OL | Advanced cultivar | Australia |  |  |  |
| FRIO | Advanced cultivar | USA |  |  |  |
| Gila | Advanced cultivar | USA |  |  |  |
| LEED | Advanced cultivar | Unknown |  |  |  |
| LESEF 174 | Advanced cultivar | Canada |  |  |  |
| SIGMA 11 | Advanced cultivar | Unknown |  |  |  |
| SIGMA 19 | Advanced cultivar | Unknown |  |  |  |
| SIGMA 24 | Advanced cultivar | Unknown |  |  |  |
| SIGMA 25 | Advanced cultivar | Unknown |  |  |  |
| SIGMA 27 | Advanced cultivar | Unknown |  |  |  |
| SIGMA 29 | Advanced cultivar | Unknown |  |  |  |
| SIGMA 38 | Advanced cultivar | Unknown |  |  |  |
| SIGMA 42 | Advanced cultivar | Unknown |  |  |  |
